# Combination of Metformin, Sodium Oxamate and Doxorubicin Induces Apoptosis and Autophagy in Colorectal Cancer Cells *via* Downregulation HIF-1α

**DOI:** 10.3389/fonc.2021.594200

**Published:** 2021-05-26

**Authors:** Jossimar Coronel-Hernández, Rebeca Salgado-García, David Cantú-De León, Nadia Jacobo-Herrera, Oliver Millan-Catalan, Izamary Delgado-Waldo, Alma D. Campos-Parra, Miguel Rodríguez-Morales, Norma L. Delgado-Buenrostro, Carlos Pérez-Plasencia

**Affiliations:** ^1^ Laboratorio de Genómica Funcional, Unidad de Biomedicina, FES-Iztacala, UNAM, Tlalnepantla, Mexico; ^2^ Laboratorio de Genómica, Instituto Nacional de Cancerología, Tlalpan, Mexico; ^3^ INCMNSZ, Unidad de Bioquímica, Tlalpan, Mexico; ^4^ Laboratorio de Toxicología, Unidad de Biomedicina, FES-IZTACALA, UNAM, Tlalnepantla, Mexico

**Keywords:** HIF-1α, autophagy, ULK1, mir-26a, AKT, proliferation

## Abstract

Colorectal cancer (CRC) is the third leading cause of cancer-related death worldwide in both sexes. Current therapies include surgery, chemotherapy, and targeted therapy; however, prolonged exposure to chemical agents induces toxicity in patients and drug resistance. So, we implemented a therapeutic strategy based on the combination of doxorubicin, metformin, and sodium oxamate called triple therapy (Tt). We found that Tt significantly reduced proliferation by inhibiting the mTOR/AKT pathway and promoted apoptosis and autophagy in CRC derived cells compared with doxorubicin. Several autophagy genes were assessed by western blot; ULK1, ATG4, and LC3 II were overexpressed by Tt. Interestingly, ULK1 was the only one autophagy-related protein gradually overexpressed during Tt administration. Thus, we assumed that there was a post-transcriptional mechanism mediating by microRNAs that regulate UKL1 expression during autophagy activation. Through bioinformatics approaches, we ascertained that ULK1 could be targeted by mir-26a, which is overexpressed in advanced stages of CRC. *In vitro* experiments revealed that overexpression of mir-26a decreased significantly ULK1, mRNA, and protein expression. Contrariwise, the Tt recovered ULK1 expression by mir-26a decrease. Due to triple therapy repressed mir-26a expression, we hypothesized this drug combination could be involved in mir-26a transcription regulation. Consequently, we analyzed the mir-26a promoter sequence and found two HIF-1α transcription factor recognition sites. We developed two different HIF-1α stabilization models. Both showed mir-26a overexpression and ULK1 reduction in hypoxic conditions. Immunoprecipitation experiments were performed and HIF-1α enrichment was observed in mir-26a promoter. Surprisingly, Tt diminished HIF-1α detection and restored ULK1 mRNA expression. These results reveal an important regulation mechanism controlled by the signaling that activates HIF-1α and that in turn regulates mir-26a transcription.

## Introduction

Colorectal cancer accounts for the fourth most common type of cancer and the third most common cause of cancer-related death worldwide in both sexes (1). Treatment for CRC depends on tumor localization and the stage of diagnosis (2). Chemotherapy is the first treatment option. 5-Fluorouracil (5-FU), as well as oxaliplatin, is the most frequently prescribed chemotherapeutic drug. However, these chemotherapeutics have several side effects, including gastrointestinal problems, myelotoxicity, cardiotoxicity, nausea, vomiting, stomatitis, gastritis, and severe diarrhea (3–5). Besides, monoclonal therapies such as Panitumumab and Cetuximab [monoclonal antibodies directed to the epidermal growth factor receptor (EGFR)] have demonstrated clinical efficacy in patients with colorectal cancer in advanced stages. Nevertheless, they also presented side effects like high degree skin toxicity (6). Moreover, approximately 50% of patients with colorectal cancer are not candidates for immunotherapy like Panitumumab and Cetuximab because they have mutations in the RAS gene (7). Several studies suggest tumor cell metabolic pathways may become potential therapeutic targets for the reduction of cell growth and progression *in vitro* promoting cell death processes such as apoptosis (8–10). Thus, cancer metabolic rewire understanding it is necessary to search for therapeutic schemes that demonstrate their efficiency in the reduction and elimination of the tumor growth, and that have the least number of side effects to the patient.

Cancer cells have a high demand of catabolites and take them up from blood circulation to maintain a reduction-oxidation balance and generate energy to proliferate and grow (11). Cancer cells predominantly use glucose to generate ATP, but the pyruvate obtained by glycolysis is not metabolized *via* the tricarboxylic acid (TCA) cycle and oxidative phosphorylation (OXPHOS), pyruvate is mainly oxidized into lactate through LDH-A overexpression (12). This molecular event is known as the Warburg effect (aerobic glycolysis). The main feature of this process is that ATP production is 100 times faster than TCA-OXPHOS in normal cells (13); thus, LDH-A represents an important therapeutic target. Sodium oxamate is an analog of pyruvate functioning as a competitive inhibitor (14, 15). Owing to this fact, our group developed a therapeutic strategy based on doxorubicin, metformin, and sodium oxamate combination. We found a fast tumor reduction and expression decrease of anti-inflammatory cytokines in the AOM/DSS CRC mouse model (16) and total tumor diminution in the MDA-MB-231 subcutaneous xenograft model (17). In both animal models, there was tumor growth arrest due to the activation of apoptosis and autophagy. Worth noting that our therapeutic proposal is lower in cost compared to immunotherapy and frequently prescribed chemotherapeutic drugs (7), offering an effective alternative, which reaches a greater number of patients. Nevertheless, the molecular mechanism has not been described yet.

Evidence suggests that microRNAs can modify the biological effect of pharmacological treatments through miRNAs-mRNA interaction, inducing chemoresistance, or chemosensitivity (18, 19). On the other hand, drug therapy could modify the expression profile of post-transcriptional regulators such as miRNAs. In breast cancer cell lines, doxorubicin altered the expression of 107 miRNAs related to cell proliferation and chemotherapy resistance (20). In this scenario, the participation of specific miRNAs has been described. An example is mir-26a, which acts as an oncomir in many types of cancer (21–24). Remarkably in CRC, mir-26a enhances proliferation (25), promotes migration (26), and regulates glucose metabolism (27). In hepatocarcinoma, restoration of mir-26a expression in cells treated with doxorubicin switched autophagy-induced chemoresistance into apoptosis through ULK1 negative regulation (28). Contrary, in human oral cancer mir-26a, increases its expression and induced apoptosis when cells are treated with metformin (29). Thus, we hypothesized that the autophagy and apoptosis caused by Tt could be mediated by mir-26a in CRC.

In this study, we showed that triple therapy induces apoptosis and autophagy by modulating ULK1 protein levels *via* miR-26 inhibition. The use of the drug combination reduced the levels of the transcriptional factor HIF-1α, having an impact on two processes: inhibition of the mTOR/AKT survival pathway and a decrease in the promoter occupancy of mir-26a by HIF-1α. Mir-26a is a widely documented oncomir; its effects on proliferation, evasion of apoptosis, and reduction of autophagy may be regulated by HIF-1α. In this study, we demonstrated that Tt induces autophagy through the reduction of mir-26a *via* HIF1-α.

## Materials and Methods

### Patient Samples and Tissue Expression for *In Silico* Meta-Analysis

Twenty CRC paraffin-embedded tissue samples staged locally advanced; ten Crohn’s disease paraffin-embedded tissue samples and 13 healthy tissues without macro and microscopic lesions were obtained by colonoscopy from INCAN (Instituto Nacional de Cancerología, Mexico) pathology registry. The investigation was approved by the ethics and scientific committee (approval number INCAN/CI/826/17). Mature mir-26a expression data was obtained from The Cancer Genome Atlas in different stages of 455 Colorectal Cancer samples and normalized with deseq2 (Bioconductor Package; data was compared with eight healthy tissues.

### Cell Culture and Transfection

CRC-derived HCT116 cells (ATCC CCL-247) were cultured in RPMI medium supplemented with 10% (v/v) fetal bovine serum and maintained (FBS) at 37°C with 5% CO2. CRC-derived SW620 (ATCC CCL-227) and non-tumoral immortalized epithelial CRL1790 colon cells obtained from ATCC were cultured in DMEM F12 medium. All employed plasmids, miRNA mimics, and inhibitors were transfected using Lipofectamine 3000 transfection agent (Invitrogen), following the manufacturer’s protocol.

### Inhibitory Concentration 50

To obtain the inhibitory concentration 50 (IC_50_) of the drugs, we followed the protocol described in (30). The cells were seeded in 96 well plates (7,000 cells per well), after 24 h, they were stimulated with the drugs at different concentrations: Doxorubicin (0.25, 0.5, 0.75, 1, 1.25, and 1.5 μM) (Doxolem ^®^RU, 10 mg/5 ml), Metformin (0.001, 0.1, 5, 20, 35, 50, 65 mM) (Santa Cruz Biotechnology) and Sodium Oxamate (0.01, 0.5, 5, 15, 25, 35, 45 mM) (Santa Cruz Biotechnology). Individual treatments were performed with their respective IC_50_ previously obtained. For the triple therapy IC_50_, we choose the IC_50_ of each drug, to recalculate a new IC_50_ in combination, taking as a start point the individual IC_50_. Cells were fixed with cold trichloroacetic acid at 10% and stained with Sulforhodamine B (MP BIOMEDICALS). The optical density was measured in a microplate reader (EPOCH, Biotek) at 510 nm. The IC_50_ was determined from a linear regression (R2 = 0.92) to obtain the gradual dose–response graphs.

### Monitoring Autophagy by GFP-LC3

For autophagy assay, transfected HCT116 cells expressing fusion protein GFP-LC3 (autophagy marker) were seeded in 6-well plates with coverslips for 24 h. The next day, drugs were added, cells were monitored at 8 and 12 h of treatment and fixed using 3.7% paraformaldehyde (PFA) at room temperature for 30 min and permeabilized with 0.5% Triton X-100 for 3 min. Then, coverslips were rinsed with PBS and were mounted with Vectashield (Vector Laboratories VECTASHIELD^®^ Antifade Mounting Medium with DAPI) and fluorescent images were acquired utilizing the Leica TCS SP8 inverted microscope. All images were processed with Leica Application Suite X.

### TUNEL Assay

Cells at 80% of confluency were treated with different combinations of drugs for 24 h. The cells were fixed with 4% paraformaldehyde and permeabilized with 0.2% Triton X-100. DNA fragmentation was determined by TdT-mediated dUTP nick end labeling (TUNEL) as described by the manufacturer (DeadEnd Fluorometric TUNEL System, Promega Part# TB235). Fluorescent images were obtained using a Leica TCS SP8 inverted microscope. All images were processed with Leica Application Suite X and Illustrator CC.

### RNA Expression Analysis

Total RNA was isolated from cultured cells (CRL1790, HCT116, and SW620) grown to approximately 80–85% confluence, using the TRIzol reagent (Invitrogen) following the manufacturer’s protocol. miRNAs were isolated from tissue blocks using the miRNeasy FFPE kit (Qiagen) following the manufacturer’s recommendations. Mir-26a and ULK1 messenger were detected in cultured cells and tissue block samples by qPCR using the Bio-Rad CFX 96 Touch and mir-26a with Taqman Universal PCR Master Mix 2 (Applied Biosystems) or the SYBR Select Master Mix for CFX (Applied Biosystems), respectively. To measure mir-26a, cDNA was generated from 100 ng total RNA with the TaqMan Micro-RNA Reverse Transcription Kit (Applied Biosystems) in a 15 µl volume; qPCR was performed using 1 µl cDNA and the mir-26a with Taqman Universal PCR Master Mix 2. Amplification conditions were 10 min at 95°C, followed by 40 cycles of 95°C for 15 s and 68°C for 60 s. For ULK1 mRNA detection (primer Fw TCATGGAGCAAGAGCACACG and primer Rv CTGCTTCACAGTGGACGACA), cDNA was synthesized from 2 µg total RNA using the High-Capacity cDNA Reverse Transcription Kit (Applied Biosystems); 1 µl of this reaction was used for qPCR. Amplification conditions were 2 min at 95°C for initial denaturation, followed by 40 cycles of 95°C for 15 s, primer-dependent annealing temperature for 15 s, and 72°C for 60 s. Relative expression data were calculated through the ΔΔCt method (Applied Biosystems) and normalized relative to U6 snRNA or GAPDH mRNA accordingly.

### Protein Expression Analysis

Protein extracts from cultured cells were obtained by homogenization in RIPA buffer (Santa Cruz Biotechnology), then cleared by centrifugation at 12,000 rpm for 20 min. For immunodetection, 50 µg total protein from tumor tissue or cultured cells were mixed with Laemmli sample buffer, boiled, separated in 12 or 15% SDS-PAGE, and transferred in a PVDF membrane (Amersham-GE Healthcare). Membranes were incubated overnight using a 1:1,000 (v/v) dilution of the anti-ULK1 (Cell Signaling), 1:3,000 of the anti-Beclin-1 (Cell Signaling), 1:3,000 of the anti-LC3 (Cell Signaling), 1:3,000 of the anti-ATG4b (Santa Cruz), and 1:3,000 of the anti-HIF-1alpha (GeneTex); for detection, 1:2,500 (v/v) dilutions of HRP anti-rabbit or anti-mouse conjugate antibodies (Santa Cruz Biotechnology) were used. Finally, using the Super Signal West Femto chemiluminescent substrate (Thermo Scientific), the membranes were scanned in the C-Digit blot scanner (Li-Cor) and the images were analyzed for densitometry in the associated Image Studio software (LiCor). Membranes were stripped and re-probed for detection of actin (anti-actin, Sc-47778) as a loading control. A representative image from three independent experiments is shown.

### Luciferase Reporter Assays

Reporter plasmids were constructed by ligation of synthetic oligonucleotide duplexes (IDT) containing putative mir-26a target regions in the ULK1 3’UTR: 5’ CTAGTCCTGAATCAGTAGATACTTGAA3’ and 5’AGGACTTAGTCATCTATGAACTTTCGA 3’ or mutant ULK1 3’ UTR target region 5’CTAGTCCTGAATCAGTAGACGTCCAGACGAA3’ and 5’AGCTTTCGTCTGGACGTCTACTGATTCAGGA3’, obtained from TargetScan (31), microRNA.org (32) and Starbase (33), forming a DNA duplex with overhanging SpeI and HindIII half-sites in the 5’ and 3’ ends respectively, which was cloned into the appropriately digested pMIR-REPORT plasmid (Ambion). This construct was co-transfected with mir-26a mirVana miRNA mimic (Applied Biosystems) and the pRL Renilla Luciferase Control Reporter (Promega) into HCT116 cells. Luciferase activity was analyzed using the Dual-Luciferase Reporter Assay System (Promega) 24 h after transfection, in a GloMax 96 Microplate Luminometer (Promega). Luciferase activity was normalized to Renilla activity for each transfected well; each experiment was performed by triplicate.

### Chromatin Immunoprecipitation (ChIP) Assay

To validate the interaction of HIF-1α with mir-26a promoter, we carried out a Chromatin Immunoprecipitation assay (Merck Millipore) following the manufacturer’s protocol. HCT116 cells were used under basal conditions and exposed to different treatments. Protein-DNA cross-linking was done with formaldehyde (1%) and was sonicated for 10 s and four pulses. Immunoprecipitation of complexes was made with anti-HIF-1a antibody (Abcam ab2185) specific for Chip assays. The immunoprecipitated DNA sequences for CTDSPL and CTDPS2 were amplified by PCR by standard conditions and then analyzed by electrophoresis on a 1.5% agarose gel (CTDSPL promoter primer sequence was Fw ACAGCACCCGAAAATGCCCAC and Rv GGATCGGGAGTGATCTGTGC and CTDSP2 promoter primer sequence were Fw AGCGGCTTGTCTTGGTCACC and CTAACAACATTCCAGGCGCCA).

### Real-Time Analysis of Cell Proliferation and Migration

The xCELLingence real-time cell analyzer (RTCA) instrument was used with E-plates to analyze the proliferation of cells transfected with mir-26a mimic and inhibitor. HCT116 cells were cultured and transfected in six well-plates (5 × 105 cells per well) with 10% FBS-supplemented medium at 37°C for 24 h, after cells were trypsinized and counted by Neubauer chamber. We plated 1 × 104 cells per E-plate well with 10% FBS-supplemented in 150 µl/well. The RTCA recorded cell index values over 24 h by 15 minute-intervals.

### Statistical Analysis

All values are expressed as the mean ± SEM. Data were analyzed in the Prism 5.0 (GraphPad) software using a one-way ANOVA analysis followed by Tukey’s Multiple Comparison Test.

## Results

### Triple Therapy Inhibits Proliferation in CRC Derived Cells

**Figure 1:**
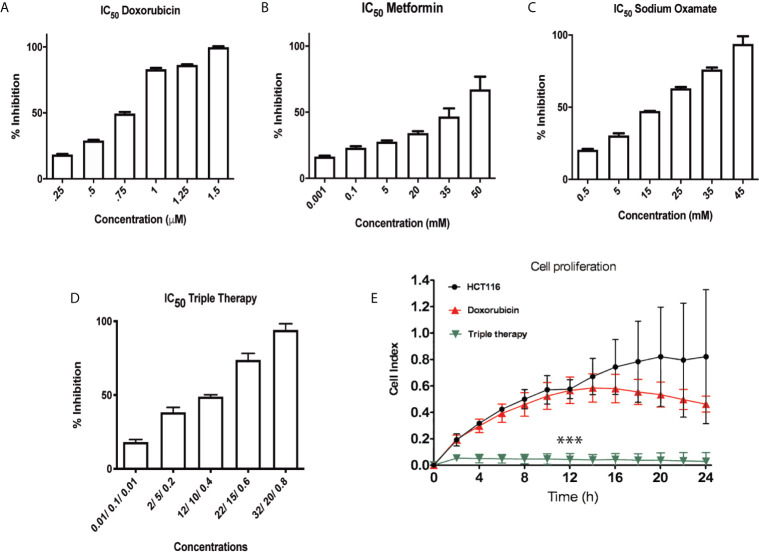
Triple therapy inhibits proliferation in a HCT116 cell line. **(A**–**C)** Inhibitory Concentration 50 (IC50) of the drugs Doxorubicin, Metformin and Sodium Oxamate in the HCT116 cell line at 24 h of exposure to these drugs. **(D)** IC50 values of the drugs in combination. **(E)** Real Time cell proliferation of HCT116 under Dox and Tt treatment (***p < 0.001).

Previously, our group showed that the pharmacological combination of doxorubicin, metformin, sodium oxamate was able to inhibit cell proliferation and to induce cellular death in both two *in vivo* models (16, 17). According to previous evidence, we hypothesized that Tt would have the same effect in CRC cells. Then, HCT116 cells were treated with Dox, Met, Ox, or Tt as were described in the methodology section. The IC_50_ values were Dox (0.75 μM), Met (20 mM), Ox (15 mM) and Tt [Dox (0.4 μM) Met (12 mM) Ox (10 mM)] (**Figures 1A–D**). As expected, the IC_50_ pharmacological concentrations were lower than the concentration of the individual drugs, suggesting a possible synergic effect. We used the xCELLingance RTCA system to measure the Tt effect in the cell proliferation for each group. Tt inhibited significantly the proliferation in HCT116 cells after the first two hours of treatment, while doxorubicin had a slight effect until 12 h after the treatment. However, in terms of the concentration used, there were no significant differences in the control (**Figure 1E**). This result suggested Tt had a direct effect on HCT116 cell proliferation.

### Triple Therapy Induces Apoptosis and Autophagy in HCT116 Cells

**Figure 2:**
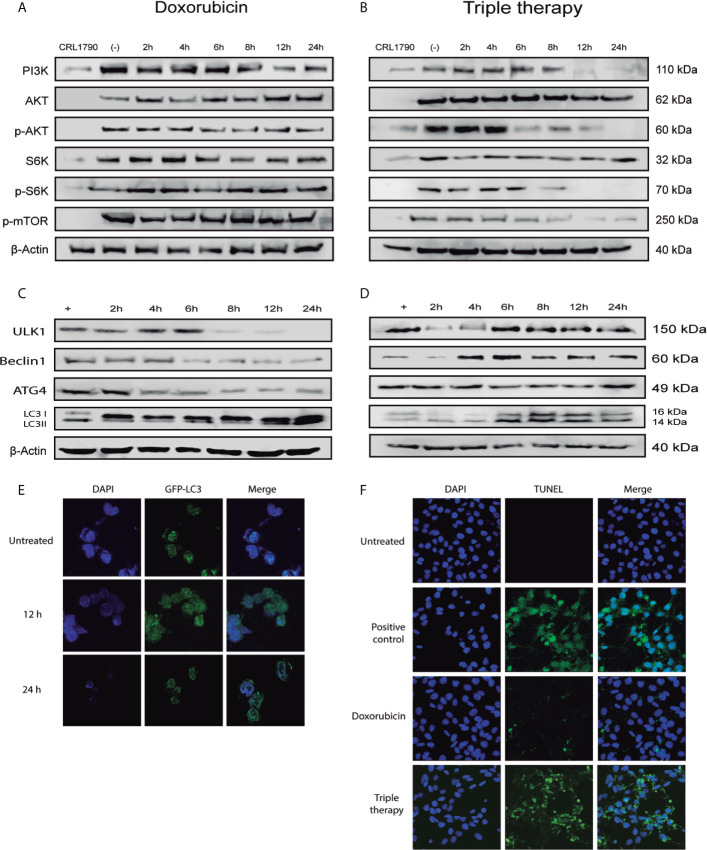
Tt induces cell death by autophagy in HCT116 cells. **(A, B)** Detection of PI3K/AKT pathway components in HCT116 cells treated with Doxorubicin and Tt, β-actin was used as loading control. **(C, D)** Protein levels of key autophagy components in HCT116 cells treated with Doxorubicin and Tt. **(E)** Confocal microscopy of HCT116 transfected with LC3-GFP under Tt treatment for 8 and 12 h. **(F)** Tunel assay of HCT116 under Dox and Tt treatment.

It has been reported that metformin and doxorubicin arrest cell cycle and induce cell death through negative regulation of PI3K/AKT pathway (34, 35). So, we measured different proteins of this pathway and found that doxorubicin did not affect the expression levels of any of these proteins (**Figure 2A**). Moreover, Tt diminished PI3K and p-AKT levels after 6 hours of treatment, and this downregulation was consistent with the decrease of downstream targets of PI3K/AKT pathway, p-S6K, and p-mTOR (**Figure 2B**) (**Supplementary Figure 1**). Such results suggest that Tt induces cell cycle arrest. To confirm whether Tt also induces cell death as it has been described in previous reports, we performed a set of western blots to detect autophagy (ULK1, Beclin 1, ATG4, and LC3II) and TUNEL assay to observe apoptotic cells. As to autophagy, an increase in ULK1, Beclin 1, ATG4, and LC3II protein detection would indicate that cells are dying in this way. Thus, we observe a slight reduction in protein levels during the treatment with doxorubicin (**Figure 2C**); showing that doxorubicin did not induce autophagy. This evidence correlates with a minor decrease in the proliferation rate. On the other hand, in the Tt group, we noticed a visible gradual increase in ULK1 levels from beginning to the end of the treatment. Also, we found high levels of Beclin 1 and LC3II from 4 h, indicating that Tt activates autophagy (**Figure 2D**). To verify autophagy induction, we evaluated autophagosome formation by LC3 localization. In control cells, LC3-GFP is localized surrounding the nucleus at basal concentration. After 12 h of treatment, LC3-GFP increased its detection, indicating autophagosome recruitment. Finally, at 24 h, LC3-GFP was concentrated in the cytoplasmatic border forming the autophagolysosomes, validating our previous results (**Figure 2E**). Finally, to confirm apoptosis, we used the TUNEL assay and noticed that doxorubicin had a minimum apoptotic effect. Interestingly, Tt promoted apoptosis in CRC cells (**Figure 2F**). These results would indicate that Tt promotes inhibition of PI3K/AKT pathway resulting in autophagy and apoptosis induction in CRC derived cells.

### mir-26a Regulates ULK1 Expression in CRC Cells

**Figure 3:**
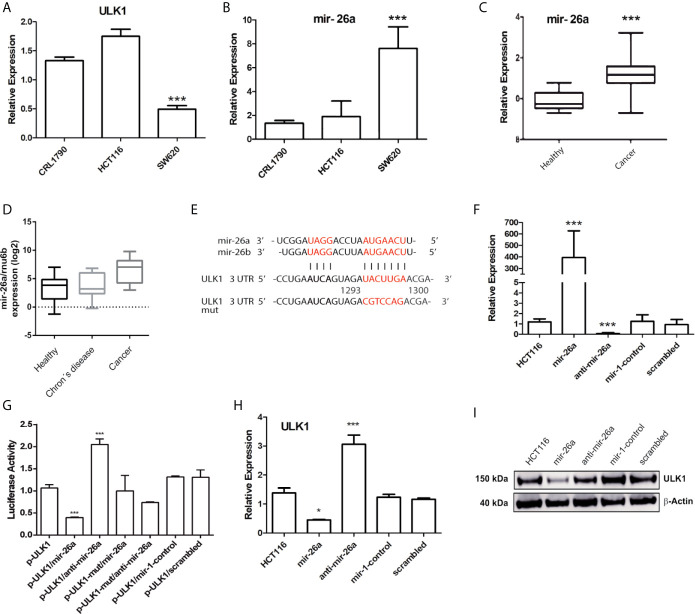
Mir-26a regulates ULK1 expression in CRC cells. **(A, B)** Expression of ULK1 and mir-26a in CRL1790 (control cells), HCT116 and SW620 (CRC cell lines) normalized with GAPDH or RNU6, respectively. **(C)** mir-26a expression of colorectal cancer samples data obtained from “The Cancer Genome Atlas” (*p < 0.05). **(D)** mir-26a expression in CRC-derived paraffin-embedded, Crohn´s disease samples and non-tumoral samples was used as a control. **(E)** Binding site between ULK1 and mir-26a predicted by bioinformatic analysis. **(F)** Expression levels of mir-26a by transfection of mir-26a mimic or inhibitor in HCT116 cells. **(G)** Luciferase activity of mir-26a/ULK1/ULK1-mut interaction region in HCT116. Mutated nucleotides are in red. **(H)** ULK1 mRNA expression and **(I)** ULK1 protein detection in HCT116 cells normalized with GAPDH and β-actin respectively. ***P < 0.001.

Previous reports have shown that metformin and sodium oxamate can induce apoptosis and autophagy concomitantly, enhancing the effect against tumor growth and proliferation (36, 37). Even though in cancer cells autophagy has been described as a protective mechanism, there is evidence that in hypoxic conditions, autophagy could mediate cell death through apoptosis activation (38). In this study, autophagy was induced by Tt for 4 h (**Figure 2D**) as well as ULK1 protein levels, a key regulator of autophagy. Next, we analyzed the expression levels of mRNA ULK1 in normal cells versus CRC derived cell. HCT116, SW620, and CRL1790 were analyzed by qPCR. We found no significant changes of ULK1 in HCT116; however, in the metastatic cell line SW620, ULK1 expression decreased by 50% (**Figure 3A**). We thought that a post-transcriptional regulation could be exerted on ULK1. Thus, a bioinformatic analysis using TargetScan, microRNA.org, and Starbase identified mir-26a as a possible ULK1 regulator. Mir-26a expression was measured in the same cell lines and no changes were observed in HCT116 versus CRL1790; meanwhile, SW620 had a seven-fold overexpression (**Figure 3B**).

To verify miR26a expression levels in CRC patient samples were increased, we analyzed The Cancer Genome Atlas data confirming cell line results (**Figure 3C**). Also, mir-26a was measured in CRC-derived paraffin-embedded, Crohn’s disease, and healthy tissue samples, observing the same trend (**Figure 3D**). These data indicated a consistent over-expression in mir-26a levels. Afterward, we want to explore if mir-26a could inhibit ULK1 mRNA. We employed the plasmid pMir-Report, which was cloned with the ULK1 3’ UTR specific wild type or mutant binding site (to form p-ULK1 or p-ULK1-mut, **Figure 3E**). **Figure 3F** displays the efficient transfection conditions of mir-26a mimic. Co-transfection of p-ULK1 and mir-26a resulted in a 60% reduction in luciferase activity, as compared with the vector control and the mutant type (**Figure 3G**). Moreover, when the cells were co-transfected with pULK1/anti-mir-26a, the luciferase activity was rescued as expected, suggesting mir-26a directly targets ULK1. To clarify the effect of mir-26a on ULK1 expression, we analyzed ULK1 mRNA and protein levels in mir-26a/anti-mir-26a transfected cells, observing a reduction of mRNA and protein levels (**Figures 3H, I**). These findings indicated that mir-26a is a *bona fide* inhibitor of ULK1 mRNA.

### Triple Therapy Decreases mir-26a Expression

**Figure 4:**
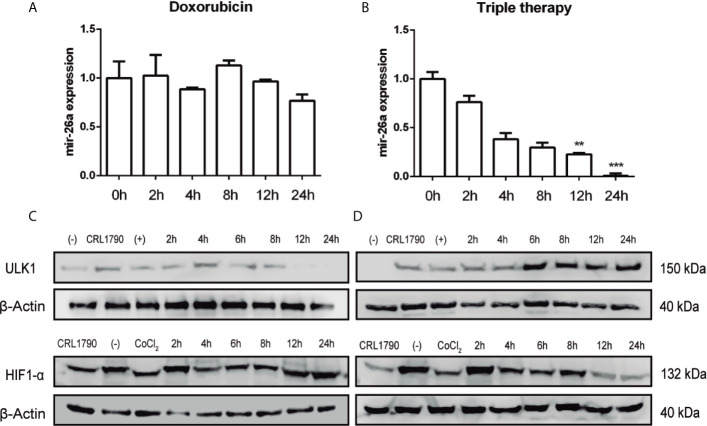
Triple therapy decreases mir-26a expression. **(A, B)** Expression of mir-26a levels in HCT116 cells treated with doxorubicin and triple therapy, respectively. **(C, D)** Protein levels of ULK1 and HIF-1α detected by western blot, β-actin was used as loading control. **P < 0.01, ***P < 0.001.

So far we have shown that in our pharmacological model, autophagy was promoted through the induction of ULK1, which is negatively regulated by mir-26a in CRC cells. Following, we want to explore if there is a correlation between mir-26a expression and Tt. To investigate the expression profile of mir-26a under Dox and Tt administration, we measured this miRNA by qPCR at 0, 2, 4, 8, 12, and 24 h after the treatment. We observed that Dox did not have any effect on mir-26a levels during the whole treatment (**Figure 4A**); meanwhile, ULK1 protein detection diminished for 8 h (**Figure 4C**). Otherwise, mir-26a decreased gradually during Tt treatment (**Figure 4B**) whereas ULK1 had a contrary effect (**Figure 4D**), showing an inverse correlation from 4 h. This result proved that Tt diminishes mir-26a levels, having a direct effect on ULK1 expression. It has been exhaustively reported that several drugs treatment alter miRNA expression profiles because drugs can directly interact with transcription factors, inhibiting their function through blocking their substrates or generating conformational changes, or marking and degrading them. HIF-1α is cataloged as a key miRNA transcriptional regulator owing to transcribing a set of specific miRNAs, knowing as *hypoxamirs*. Currently, some studies have evidenced that mir-26a belongs to this set of miRNAs (39–42). So, we rationalized that Tt could be regulating mir-26a expression *via* HIF-1α degradation. Then, we detected it at 0, 2, 4, 8, 12, and 24 h of treatment and observed that Dox did not affect HIF-1α levels (**Figure 4C**) (**Supplementary Figure 2**), correlating with mir-26a data levels (**Figure 4A**). On the other hand, Tt decreased HIF-1α detection from 4 h of treatment; however, the greatest effect was observed at 12 and 24 h (**Figure 4D**), correlating with mir-26 reduction and ULK1 increase (**Figure 4B**). These results suggested that Tt promotes an increase in ULK1 levels, modulating mir-26a expression *via* downregulation of HIF-1α.

### HIF-1α Is a Key Regulator of mir-26a Expression

**Figure 5:**
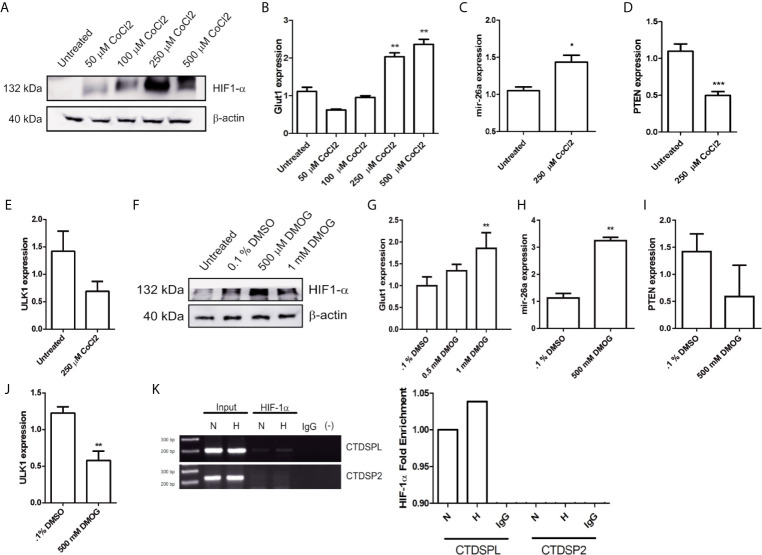
HIF-1α promotes mir-26a expression. **(A)** Protein detection of HIF-1α by western blot in HCT116 cells treated with 50, 100, 250 and 500 μM of CoCl_2_ at 24 h. **(B)** Glut1 expression in cells treated with different concentrations of CoCl_2_. **(C**–**E)** Expression of mir-26a, PTEN and ULK1 in cells treated with CoCl_2_ (250 μM). **(F)** Detection of HIF-1α by western blot after DMOG treatments. **(G**–**J)** expression of GLUT1, mir-26a, PTEN and ULK1 treated with DMOG. **(K)** ChIP assay of HIF-1α in CTDSPL and CTDSP2 promoter under CoCl_2_.

To confirm whether ULK1 expression is affected by Tt treatment *via* HIF-1α, we designed a set of experiments with two different HIF-1α stabilization models. CoCl2 binds to the HIF-1α PAS domain and Dimethyloxalylglycine (DMOG), which is a competitive inhibitor of prolyl hydroxylases resulting in HIF-1a stabilization. Hence, we detected HIF-1α by western blot in HCT116 cells treated with 50, 100, 250, and 500 mM of CoCl2 at 24 h. We observed the best effect with 250 and 500 mM (**Figure 5A**). Also, we measured Glut1 mRNA expression (a well-known target of HIF-1α) and noticed a correlation between Glut1 expression and CoCl2 administration (**Figure 5B**), thereby we used 250 mM for the following experiments. CoCl2 increased mir-26a expression (**Figure 5C**) and reduced PTEN mRNA expression (a broadly reported mir-26a target) (**Figure 5D**). Respecting ULK1, a slight decrease happened when HIF-1α was activated. Later, treatment with DMOG (500 mM) resulted in the stabilization of HIF-1α and Glut1 overexpression (**Figures 5F, G**). Similarly, mir-26a was overexpressed (**Figure 5H**) and PTEN reduced (**Figure 5I**). Regarding ULK1 was downregulated significantly with the activation of HIF-1α by DMOG (**Figure 5J**), these results showed that HIF-1α stabilization increased mir-26a expression and ULK1 mRNA down-regulation.

**Figure 6:**
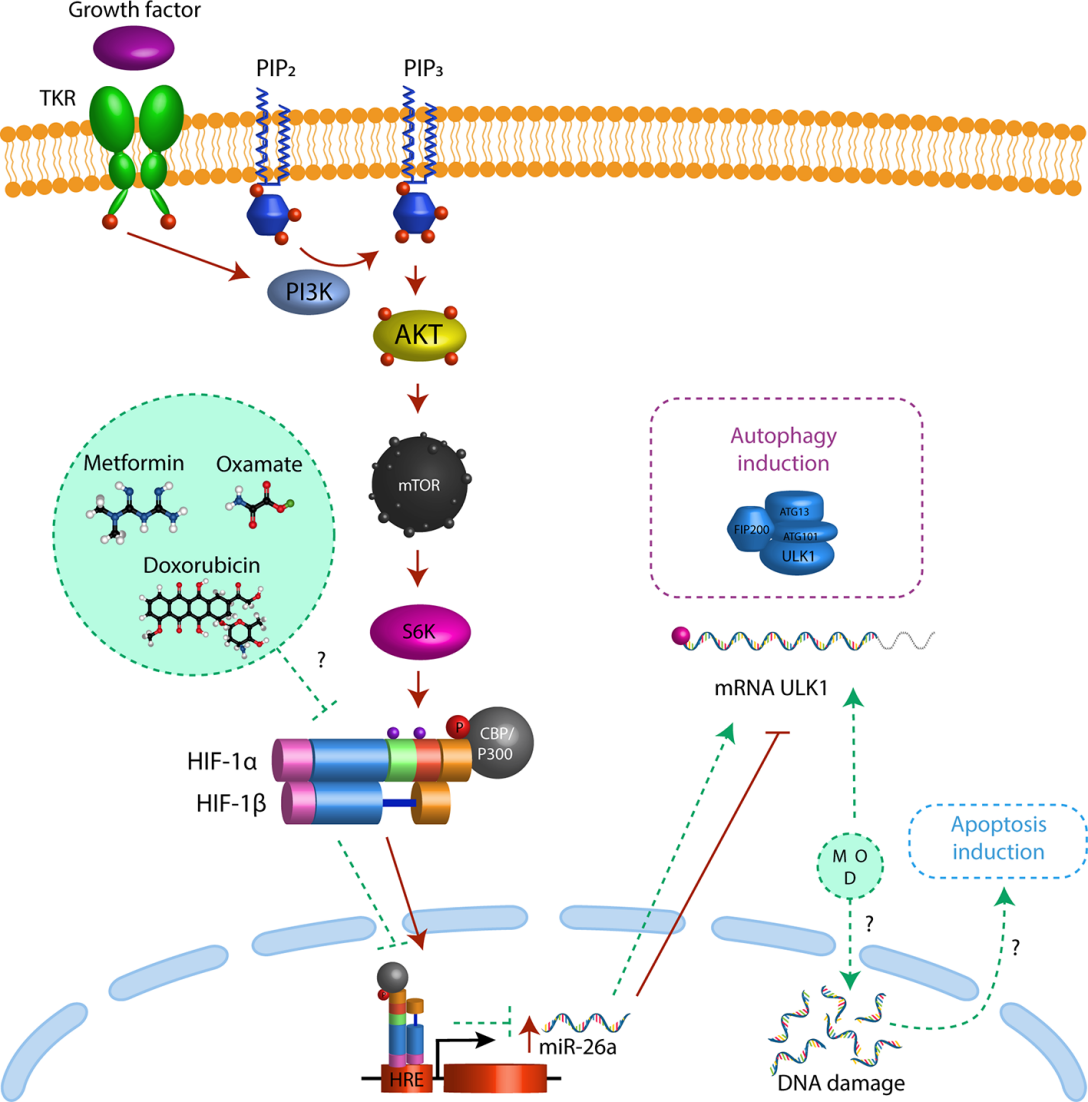
Triple therapy induces autophagy through mir-26 inhibition. Triple therapy (metformin, sodium oxamato and doxorubicin (green circle) diminish the levels of HIF1α (green dash line), resulting in a decrease in mir-26a expression (green dash line) and the release of ULK1 inhibition driving to autophagy (purple dash rectangle) and apoptosis induction (blue dash rectangle). Besides in cancer cells the PI3K-Akt pathway regulates HIF1α expression by activation of mTORC1 (red arrows), promoting their translocation to nucleus, resulting in over-expression of mir-26a (red arrow) inhibiting the mRNA translation of ULK1 (red dash line) and autophagy and apoptosis.

To confirm that HIF-1α induces mir-26a expression, we performed a ChIP assay. As mir-26a is encoded in two loci: mir-26a-1 is localized in chromosome 3 within the intron region of CTDSPL gene and mir-26a-2 is in chromosome 12 within the intron region of CTDSP2. Then, we designed two different sets of primers (one for each locus) localized 200 bp upstream and downstream of the Hypoxic Response Element located in the promoter region of both genes. We found that HIF-1α was enriched only in the promoter of CTDSPL and this interaction was enhanced by hypoxic condition (**Figure 5K**). For the first time, we showed that HIF-1α only activates mir-26a in CTDSPL in CRC. In conclusion, triple therapy abolishes proliferation and induces apoptosis and autophagy in CRC cells through induction of ULK1 which is regulated by the mir-26a/HIF-1α axis (**Figure 6**).

## Discussion

5-Fluorouracil and Oxaliplatin are the best characterized pharmacological treatments in CRC, however, chemoresistance is the principal reason for the treatment failure. In the last years, many drugs have been tested with the purpose of change conventional chemotherapy, such as doxorubicin. We previously reported that doxorubicin reduces tumor growth in the CRC mouse model, though this tumor reduction was enhanced by sodium oxamate and metformin (16). In our current work, we observed the same experimental results, doxorubicin had a slight anti-growth effect and our Tt abolished proliferation for 2 h. The combinatory effect of Tt has not been described yet, but the individual activity of each drug. Doxorubicin administration inhibited tumor growth in the SW480 CRC xenograft model (43) and stopped cell cycle progression in CT-26 murine CRC cells (44). On the other hand, metformin also has a tumor suppressor effect, decreasing oxygen consumption, and activating AMPK-mediated pathways, inducing growth inhibition and apoptosis in CRC cell lines (45, 46). Moreover, it has not reported the effect of Sodium Oxamate in CRC cells, but, in nasopharyngeal carcinoma, Ox induced cell cycle arrest and apoptosis by inhibition of LDH-A (47), promoting autophagy in gastric cancer cells to modify Akt-mTOR pathway.

All these drugs are potential individual CRC treatments, but the failure as individual therapy is the chemoresistance. It has been reported that cancer cells can develop chemoresistance to Dox (48, 49) and that the chemoresistance acquisition is associated with dysregulation of energy metabolism, hypoxia (50), and high rates of glycolysis (51). Thus, to avoid chemoresistance in CRC cells, Tt was targeted to aberrant tumoral metabolism through the decrease of lactate production by oxamate and the decrease of ATP production by metformin, resulting in apoptosis/autophagy induction. Although there is a direct correlation between chemoresistance and survival by autophagy activation (52), we observed that induction of autophagy by Tt had an anti-tumoral role, suggesting that the success of Tt is due to repressing DNA replication by doxorubicin and targeting aberrant metabolism with Ox and Met (**Figure 6**). Even though the range of concentrations of the different drugs used is extremely narrow, it was found that the combination of drugs induces apoptosis and autophagy in CRC-derived cells.

During the autophagy induction, the formation of two key protein complexes are necessary, the Atg1/ULK1 and Beclin-1/Vps34, and by blocking them using compounds as rosiglitazone resulting in autophagy reduction as was observed in experimental traumatic spinal cord injury (53). Several studies reported that their downregulation *via* microRNAs could inhibit autophagy progression in many types of cancer (54–56). Specifically, mir-26a avoids autophagy in two ways. The first one is through Beclin-1 mRNA interaction (57) and the second one is binding to mRNA of ULK1/ULK2 (58–60). Nevertheless, the effect of autophagy repression is controversial; it can act as a suppressor of cancer progression or in the maintenance of several traits of tumoral phenotype. In prostate cancer cells, autophagy is increased due to ULK1/2 overexpression, so, these cells are resistant to different drug treatments, but, when mir-26a is overexpressed, these cells get sensitized (61). Meanwhile, in hepatocellular carcinoma, the downregulation of mir-26a expression by Dox treatment activates autophagy, allowing chemoresistance (28). Besides, metformin treatment in breast cancer can increase mir-26a levels, inducing cell cycle arrest, and apoptosis through EZH2 regulation (62). Thus, the biological role of the mir-26a/autophagy axis seems to be a double-edged sword, where the elemental process to carry out cell death by these treatments is the apoptosis induction. Here we found that Tt diminished mir-26a expression, suggesting that the key component of pharmacological combination could be involved in the unbalance of energetic metabolism by oxamate and metformin.

Apoptosis and autophagy have different morphological characteristics and support diverse physiological processes, but both pathways maintain a complex connection. For instance, in some cases, these processes exert synergistic effects while in others, autophagy is activated when apoptosis is suppressed (63). Even though these two mechanisms of cell death are different, several studies have found molecules that function as regulators of both processes (64). A clear example is the proteins of the Bcl-2 family, which not only participate in the regulation of apoptosis but also as inducers or inhibitors of autophagy (65) together with Beclin-1. Under normal conditions, the BH3 domain of Beclin-1 is inhibited due to its binding with Bcl-2 or Bcl-XL inducing autophagy and apoptosis (66, 67). Also, Beclin-1 promotes apoptosis *via* Caspase 9 (68). PI3K/Akt/mTOR is also related to the interconnection between apoptosis and autophagy, it has been seen that AKT inhibits apoptosis through the phosphorylation of Bad and mTOR, inhibiting autophagy (69, 70).

Anticancer treatments could induce apoptosis and autophagy simultaneously; however, this does not indicate that both mechanisms are working together. For example, Timosapanin AIII induces apoptosis and autophagy concurrently in melanoma cells, nevertheless, autophagy is activated to counteract cytotoxicity, growth inhibition, and apoptosis induction, so, when autophagy was abolished with 3-methyladenine, the apoptotic ratio significantly raised, indicating that autophagy can antagonize the apoptosis (71). The same evidence was observed with erianin and salinomycin administration in osteosarcoma cells (72, 73) and metformin in esophageal squamous cell carcinoma (36). On the other hand, AKT-mTOR signaling pathway inhibition by bavachalcone can lead to the death of carcinoma cells by autophagy, triggering apoptosis induction (74). We found a high decrease in AKT and mTOR phosphorylation levels, indicating that autophagy also contributes to programmed cell death. In the same manner, when the mTOR/SGK1 axis is blocked and colon cancer cells are stressing with ROS accumulation, this could induce autophagy to precede apoptosis, so one process depends on each other (75, 76). Our data showed that sustained autophagy by the Tt treatment entails apoptosis.

A consequence of the Warburg Effect is HIF-1α stabilization, which induces angiogenesis and metastasis and could promote chemoresistance. HIF-1α also can modify miRNA expression profiles, so the group of miRNAs that are regulated by hypoxic conditions is called “*hypoxamirs*” (77). These miRNAs are dependent on oxygen levels and are transcribed directly by HIF-1α through direct binding to the Hypoxic Response Element sequence in their promoter region (78), for instance, the mir-26a. In HT29 cells, the ChIP assay demonstrated dynamic recruitments of HIF-1α to mir-26a promoter upon hypoxia exposure (39). On the other hand, experiments of overexpression of stable isoforms of HIF-1α induce overexpression of mir-103, mir-210, mir-213, mir-26a, and mir-181 by binding directly and transactivate HREs in the region promoters, validating by ChIP (41).

Thus, we described that in CRC cells, mir-26a expression depends on HIF-1α induction, affecting ULK1 mRNA expression. But Tt abolished HIF-1α stabilization in a non-described molecular mechanism yet. However, the comprehension of this mechanism could help us to develop better strategies in CRC treatments.

## Data Availability Statement

The raw data supporting the conclusions of this article will be made available by the authors, without undue reservation.

## Ethics Statement

Current investigation was approved by Ethics and Scientific Committee with the approval number INCAN/CI/826/17. The patients/participants provided their written informed consent to participate in this study.

## Author Contributions

Conceptualization, JC-H, RS-G, and CP-P. Software, JC-H, ID-W, ND, and OM-C. Investigation, JC-H, RS-G, MR-M, and NJ-H. Writing—original draft preparation, JC-H, RS-G, and CP-P. Writing—review and editing, JC-H, CP-P, and AC-P. Supervision, CP-P and DC-L. Project administration, NJ-H and CP-P. Funding acquisition, CP-P. All authors contributed to the article and approved the submitted version.

## Funding

JC-H is a doctoral student from Programa de Doctorado en Ciencias Biomédicas, Universidad Nacional Autónoma de México (UNAM) and received fellowship 402278 from CONACYT and 2019ND0005-11 from COMECYT. This study was supported by UNAM PAPIIT-IN231420 research funds granted to CP-P.

## Conflict of Interest

The authors declare that the research was conducted in the absence of any commercial or financial relationships that could be construed as a potential conflict of interest.

The handling editor declared a shared affiliation with the authors at the time of review.
