# Supplementary material for: Combination of Metformin, Sodium Oxamate and Doxorubicin Induces Apoptosis and Autophagy in Colorectal Cancer Cells via Downregulation HIF-1α
Source: Front Oncol. 2021 May 26;11:594200. doi: 10.3389/fonc.2021.594200 (PMC8187873; doi:10.3389/fonc.2021.594200)
Supplement: Supplementary file 1 [file DataSheet_1.pdf]

Supplementary Material

Supplementary Figures

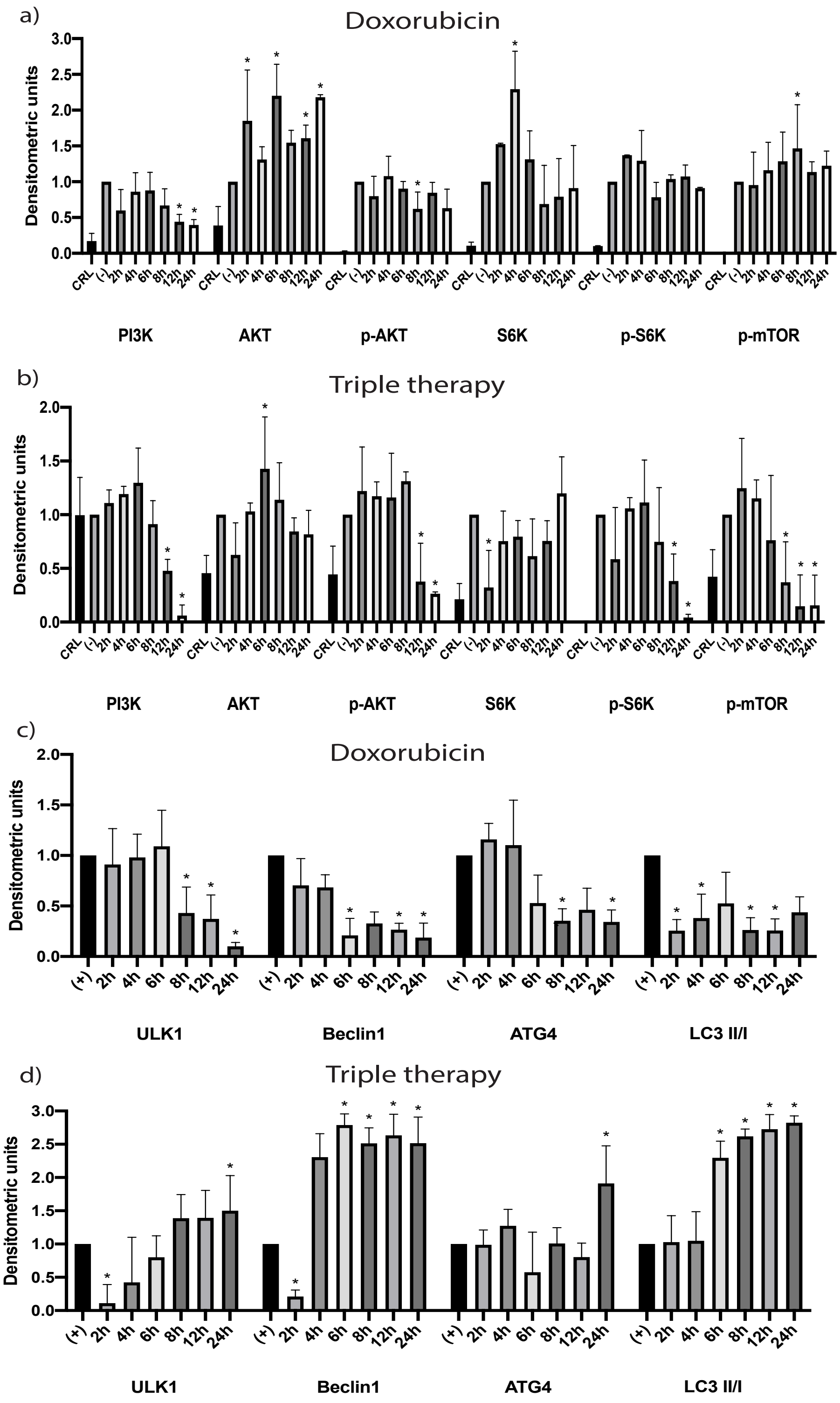

Supplementary Figure 1. Densitometry graphs of the proteins involved in PI3K/AKT pathway and in the process of autophagy in HCT116 cell line. a) and c) Doxorubicin: b) and d) triple therapy respectively. Data are presented as means ± SD. $^{*}p<0.05$. Cells treated with triple therapy decreases overtime in the PI3K/AKT pathway, while the detection of autophagy proteins increases after treated with triple therapy.
