# Supplementary material for: Combination of Metformin, Sodium Oxamate and Doxorubicin Induces Apoptosis and Autophagy in Colorectal Cancer Cells via Downregulation HIF-1α
Source: Front Oncol. 2021 May 26;11:594200. doi: 10.3389/fonc.2021.594200 (PMC8187873; doi:10.3389/fonc.2021.594200)
Supplement: Supplementary file 2 [file DataSheet_2.pdf]

Supplementary Material

Supplementary Figures

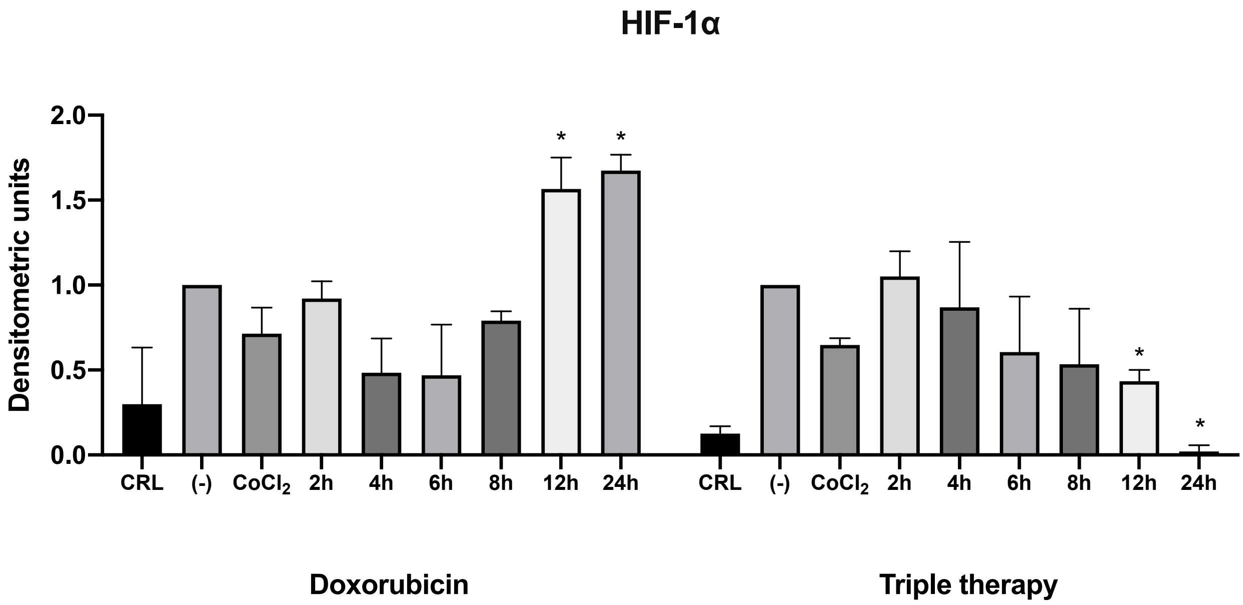

Supplementary Figure 2. Densitometry graph of HIF-1α in HCT116 cell line. Data are presented as means ± SD. *$p<0.05$. Cells treated with doxorubicin increases the detection of HIF-1α, while the detection of the same protein decreases after treated with triple therapy.
